# Successful auto‐transplantation of a mandibular third molar: A case report on restoring esthetics and function in a 66‐year‐old patient

**DOI:** 10.1002/ccr3.8911

**Published:** 2024-05-22

**Authors:** Mohsen Aminsobhani, Maryam Babaahmadi

**Affiliations:** ^1^ Department of Endodontics, Dental Research Center AJA and Tehran University of Medical Sciences Tehran Iran; ^2^ Department of Endodontics, School of Dentistry Tehran University of Medical Sciences Tehran Iran

**Keywords:** autogenous tooth transplantation, clinical dentistry, cracked tooth, root canal therapy

## Abstract

**Key Clinical Message:**

This article describes a successful case of auto‐transplantation of a mandibular third molar to replace a non‐restorable second molar, highlighting the efficacy of this procedure in restoring function with factors like asepsis, surgical technique, and postoperative care contributing to the success.

**Abstract:**

This case report describes successful auto‐transplantation of a mandibular third molar to replace a non‐restorable second molar in a 66‐year‐old patient. The procedure involved atraumatic extraction, repositioning, and stabilization of the donor tooth, followed by postoperative care and 1‐year follow‐up. The favorable outcome highlights the potential of mature third molar transplantation as an effective approach for replacement of missing or non‐restorable permanent molar teeth to restore esthetics and function. The success of the procedure was attributed to factors such as asepsis, atraumatic surgical technique, preservation of the periodontal ligament (PDL) vitality, minimal extraoral time, optimal occlusion, and adequate fixation. At the 1‐year follow‐up, the patient was asymptomatic with stable occlusion, highlighting the optimal efficacy of the procedure.

## Graphical abstract

The initial radiography and one‐year follow‐up of the patient indicate the successful outcome of the autotransplantation method.

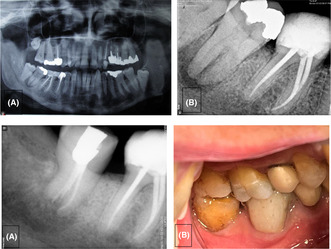

## 1 INTRODUCTION

The teeth are designed to endure high masticatory forces; however, they can still experience mechanical failure, such as incomplete fractures that may not be immediately visible^1^ but can cause pain on biting, which stops once the pressure is withdrawn, thermal sensitivity to cold, and discomfort.^2^, ^3^ The available treatment options for such fractures may include restoration, root canal therapy, or tooth extraction, depending on the extent and severity of the fracture.

Several treatment options are available for replacement of the lost teeth such as osseointegrated dental implants, fixed or removable prostheses, orthodontic space closure, and dental auto‐transplantation. Each option has its own indications and contra‐indications, and recommended timing for execution.

Dental auto‐transplantation, also known as autogenous tooth transplantation (ATT), is a procedure that involves extraction and repositioning of a donor tooth from one site to another in the same patient. This can include the transfer and implantation of impacted, embedded, or erupted teeth in the extraction socket or surgically prepared sockets. Recent advances in protocols and enhanced understanding of the physiological impact of the periodontal ligament (PDL) on the healing process have resulted in a growing interest in this dental procedure.^4^, ^5^, ^6^

The success rate of ATT varies depending on factors such as the patient's age, donor tooth type, and the adopted surgical technique.^7^ Successful ATT improves esthetics, proprioception, dentofacial development, and osteoinduction, reestablishes a stable occlusion, enhances mastication, speech, functional adaptation, and dental arch integrity, and is cost‐effective.^4^, ^8^, ^9^, ^10^ However, the success rate and applicability of ATT may vary depending on the case and the need for additional dental work. Other treatments, such as dental implants, may be more widely accepted by dental professionals and may have a higher success rate, but they may be more complex and costly than ATT.

This case report describes successful auto‐transplantation of a mandibular third molar to replace a neighboring non‐restorable second molar, with a comprehensive assessment over a 1‐year period to assess the efficacy and long‐term outcome of this innovative approach.

## 2 DETAILED CASE DESCRIPTION

**FIGURE 1:**
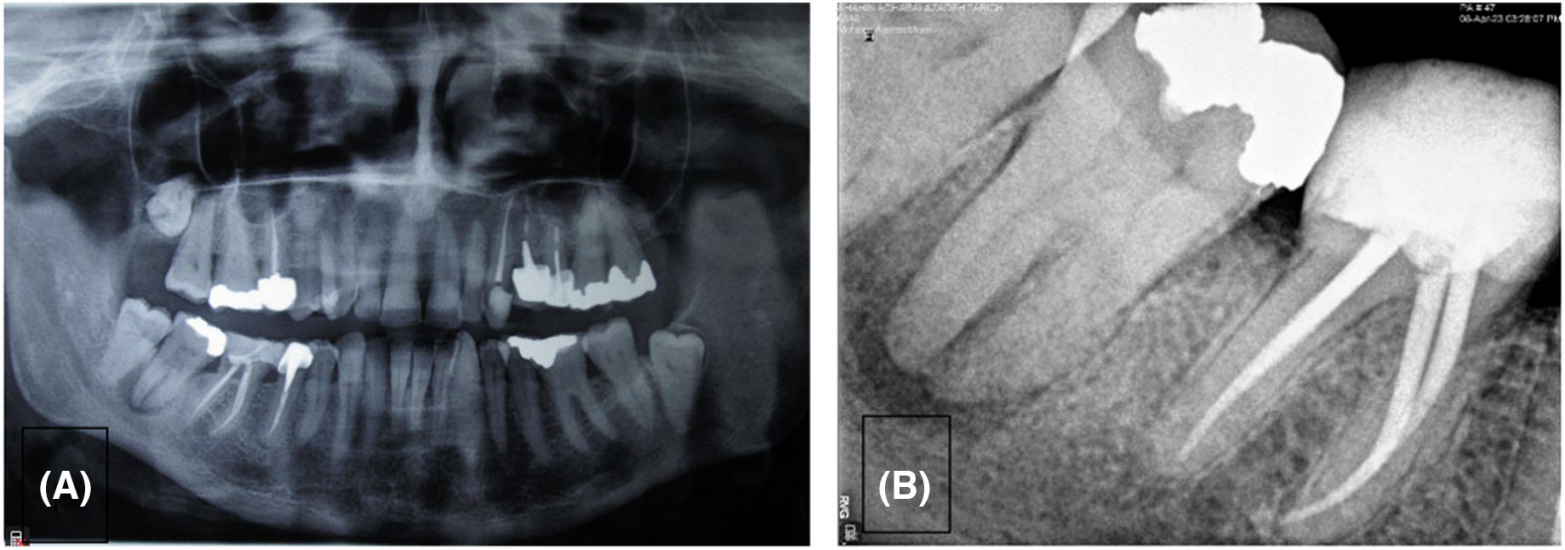
Panoramic view (A) and periapical radiograph (B) showing the periapical lesion.

**FIGURE 2:**
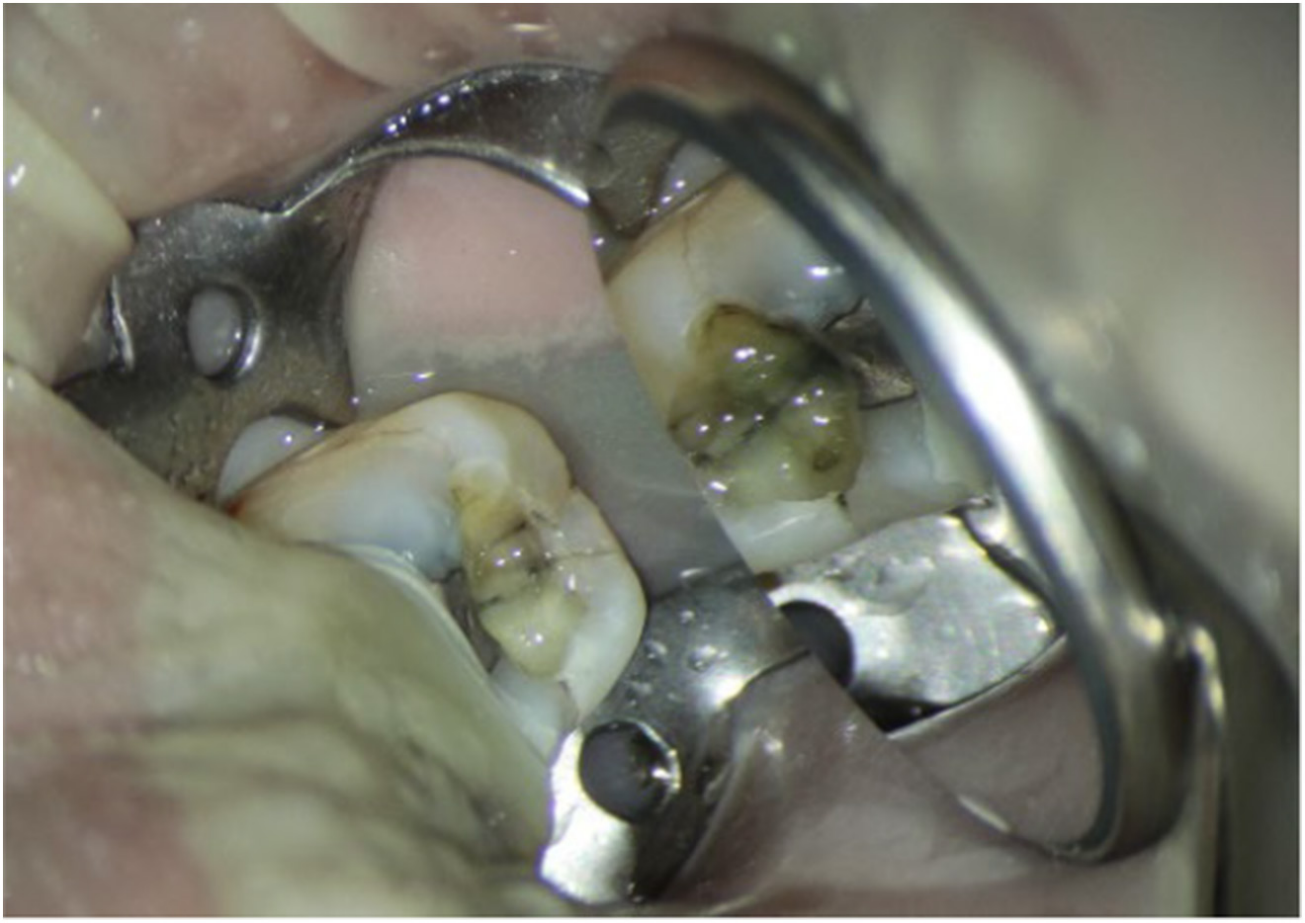
Crack line with a wide mesiodistal extension.

**FIGURE 3:**
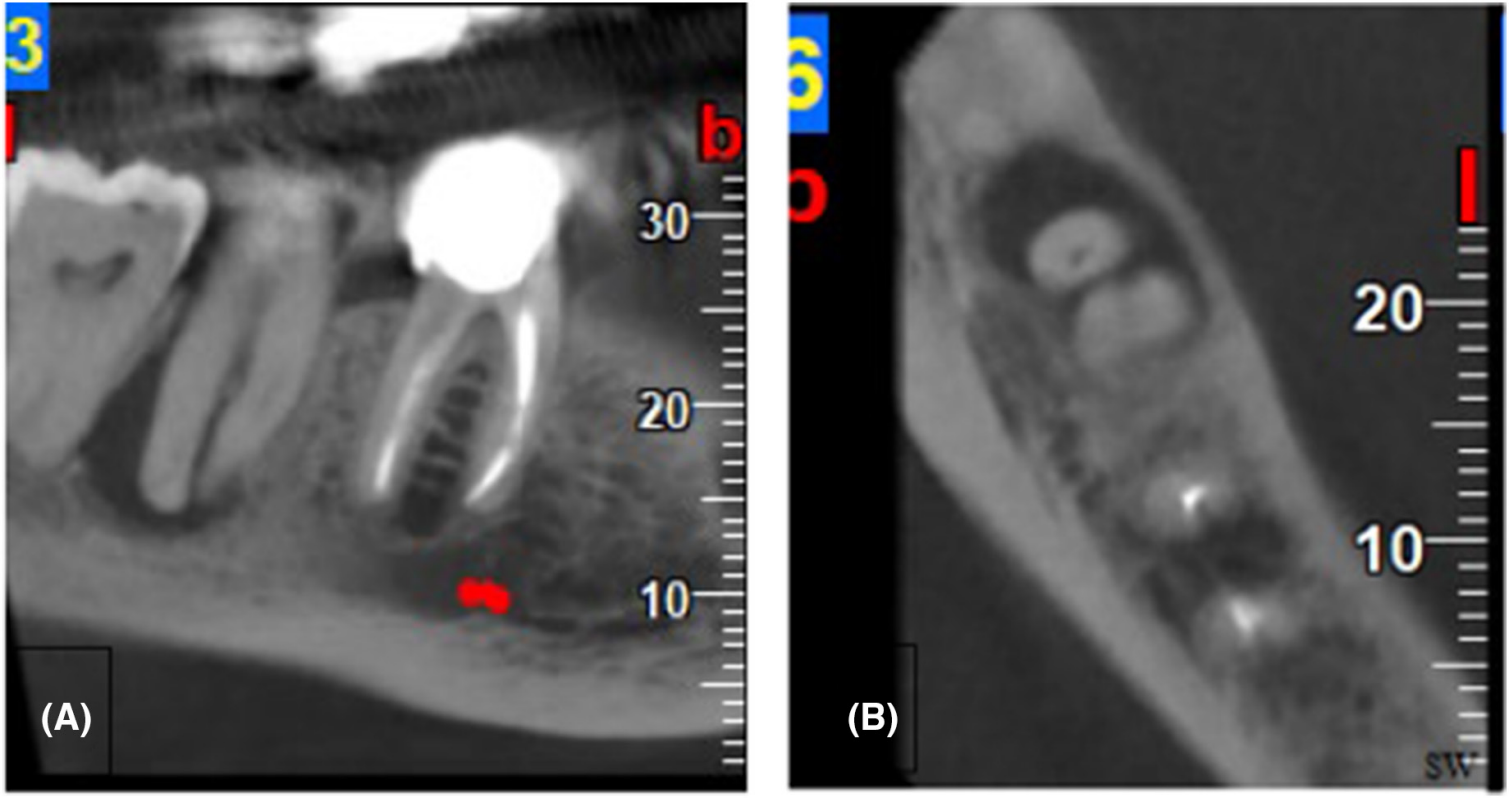
Panoramic‐like reconstructed image (A) and axial view of cone‐beam computed tomography (B) showing a J‐shaped radiolucent lesion.

A 66‐year‐old female patient with no remarkable medical history was referred to the Endodontics Department of the Faculty of Dentistry complaining of severe pain and discomfort while chewing on her mandibular left second molar. Clinical examination revealed pulp necrosis according to the negative pulp vitality tests, no sensitivity to percussion, and normal probing depth. Radiographic examination revealed a radiolucent lesion at the root apex (Figure 1). Thus, it was decided to remove the existing restoration to assess the condition of the mandibular second molar. After removal of the amalgam restoration, inspection of the pulp chamber floor under magnification with a dental microscope revealed a crack line with a wide mesiodistal extension in the pulp chamber floor extending deeply to the apical region (Figure 2). To ensure that the crack had extended to the root, a cone‐beam computed tomography scan was requested, which revealed that the crack was extensive, and the tooth could not be restored (Figure 3). Further examination revealed that the adjacent fused‐root third molar was in excellent condition. It was fully developed and was well‐positioned in the dental arch, making it an ideal candidate for ATT. As a result, the patient was thoroughly informed about the need for extraction of her mandibular second molar and the option of using the third molar for ATT, with its pros and cons. Subsequently, with the patient's consent, the decision was made to extract the mandibular second molar and replace it with the adjacent third molar through auto‐transplantation.

**FIGURE 4:**
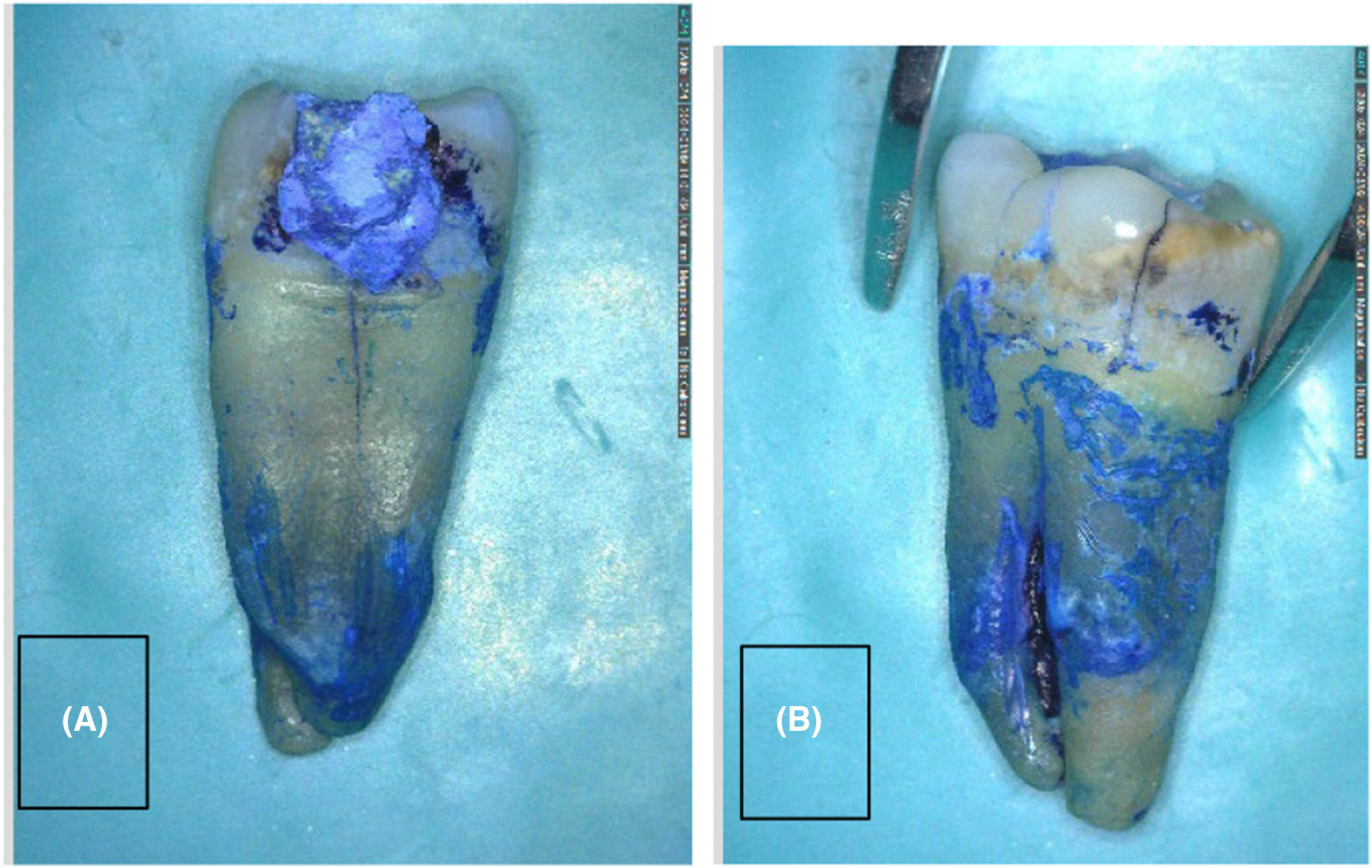
Inspection of the extracted second molar under magnification with a dental microscope following its staining with methylene blue revealed a fracture line with vertical extension.

**FIGURE 5:**
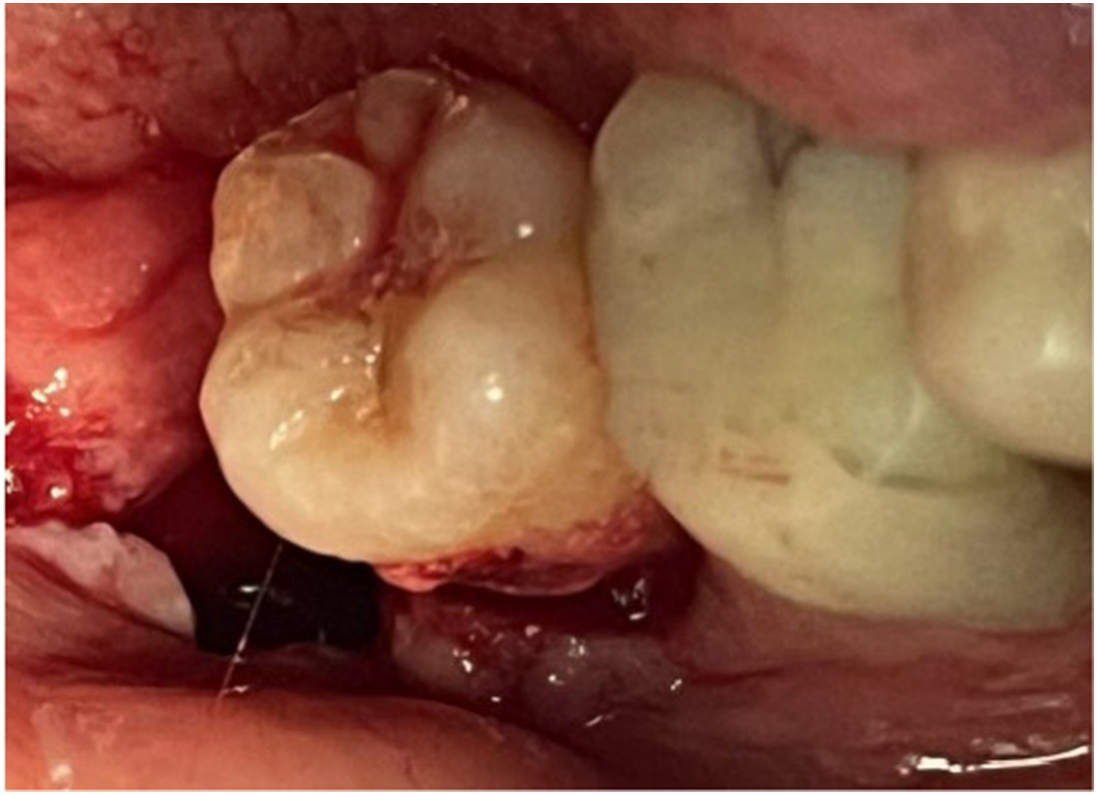
Show the transplanted molar in the 90 degrees rotated position from an occlusal view.

**FIGURE 6:**
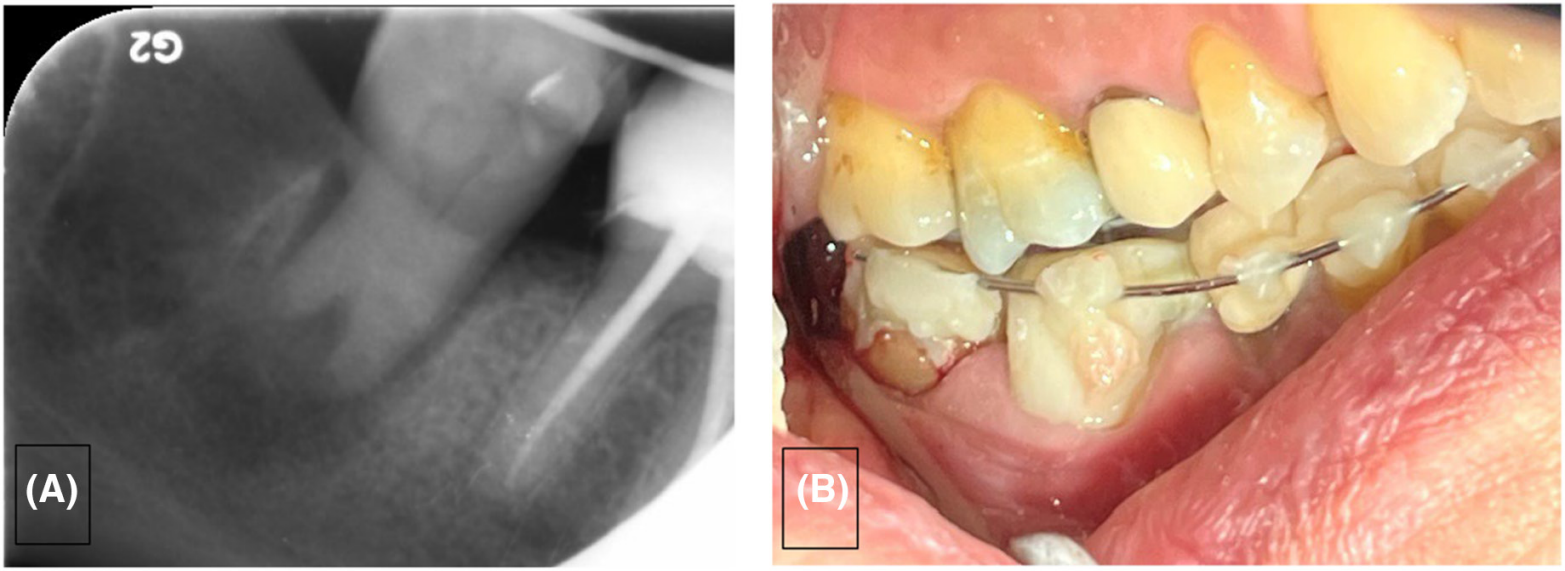
Periapical radiograph (A) and photograph image (B) taken immediately after the transplantation procedure shows the immediate post‐transplantation state of the third molar tooth.

Prior to the procedure, the patient was asked to rinse 0.2% chlorhexidine gluconate solution for the purpose of disinfection. Local anesthesia was administered through an inferior alveolar nerve block using 2% lidocaine with 1:80,000 epinephrine. The procedure was commenced with atraumatic extraction of the third molar, which was then immersed in saline solution for the shortest possible time. The unrestorable second molar was then atraumatically extracted (Figure 4). The third molar tooth was rotated by 90 degrees (Figure 5) and placed in the second molar extraction socket for more accurate adaptation to the socket walls. Subsequently, it was stabilized and fixed to the adjacent tooth using semi‐rigid orthodontic wire with 0.7 mm diameter (Figure 6); occlusal interferences with the opposing tooth were also eliminated. The transplanted third molar was further stabilized by suturing using No. 3 silk sutures to prevent its vertical movement. The entire procedure was accomplished within 5 min (from extraction to transplantation).

The patient diligently attended the follow‐up appointment scheduled after 1 day to ensure a successful postoperative recovery. The patient was advised to practice oral hygiene and use 0.2% chlorhexidine mouthwash. At the 14‐day follow‐up, the dental splint was removed, and the transplanted tooth exhibited no significant mobility. However, the pulp vitality tests indicated a lack of response; thus, non‐surgical root canal therapy was initiated.

**FIGURE 7:**
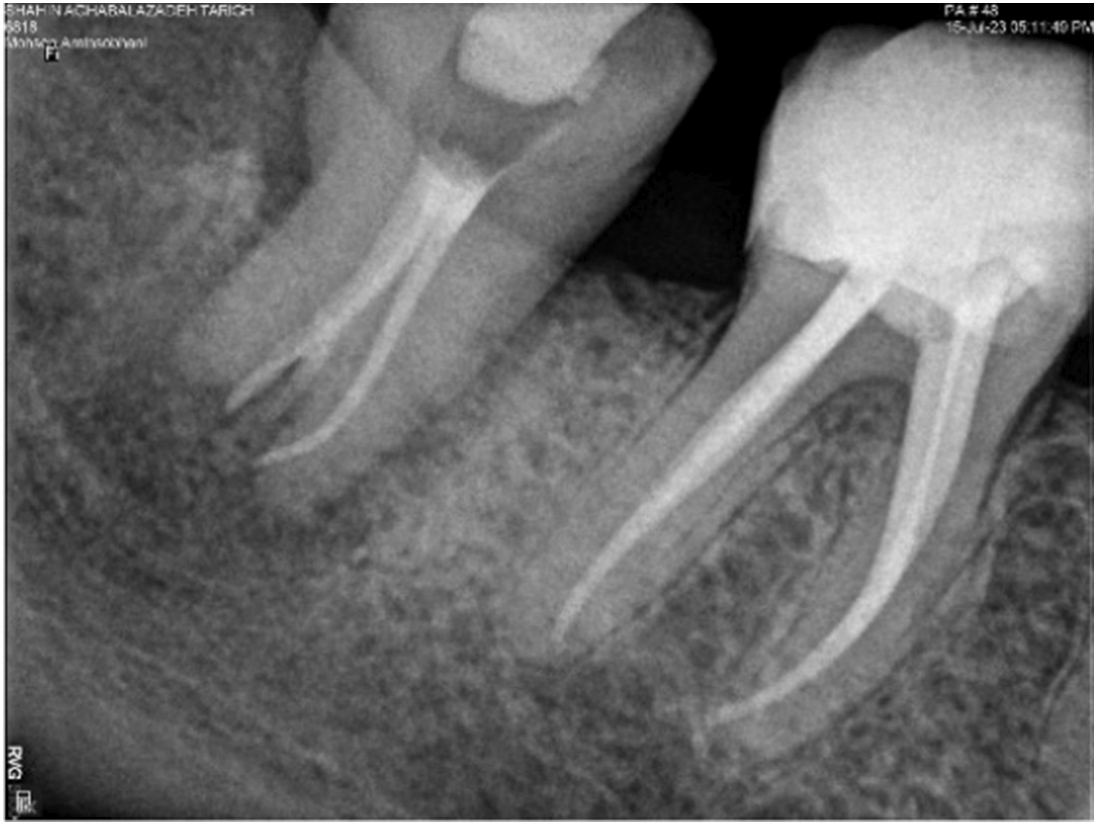
Periapical radiograph: post‐op.

**FIGURE 8:**
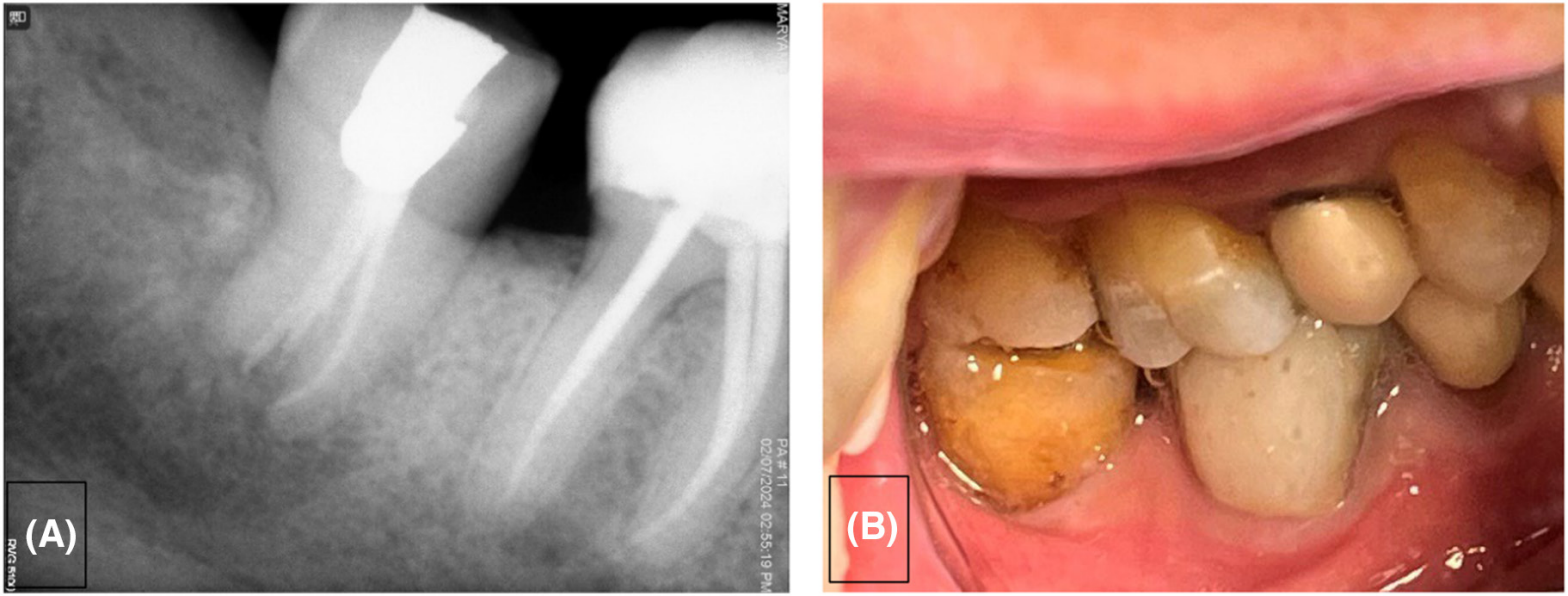
Periapical radiograph taken 1 year after transplantation illustrates significant bone and PDL regeneration with no evidence of root resorption (A), photography image taken 1 year after transplantation (B).

In the first treatment session, the pulp tissue was removed, and the canals were meticulously prepared and irrigated with saline and 5.25% NaOCl using a side‐vented 30‐gauge irrigation needle. Subsequently, a creamy mixture of calcium hydroxide and saline was introduced into the canals. After 2 weeks, the intracanal medicament was removed, and the root canals were dried with paper points and obturated with 0.04 tapered gutta‐percha points (Meta BioMed) and EndoSeal MTA root canal sealer (Maruchi USA) using the single‐cone obturation technique. The tooth was then temporarily restored with polymer‐reinforced zinc oxide eugenol restorative material (IRM) for 1 month (Figure 7). Subsequently, after ensuring the resolution of all symptoms, the tooth was permanently restored with an amalgam build‐up restoration. The decision to postpone crown placement was made due to the conservative size of the access cavity preparation and the robustness of the remaining tooth structure. The patient was recommended to maintain a soft food diet. The patient attended the scheduled follow‐up appointments at 6 months and 1 year, which included both clinical and radiographic examinations. The postoperative period was uneventful. Radiographic and clinical examinations at the 1‐year follow‐up revealed that the transplanted tooth was in normal occlusion, and had physiological mobility and effective masticatory function (Figure 8). Periodontal probing indicated no pocket or pathologies, and the satisfied patient remained symptom‐free. Bone formation around the roots was observed 1 year after the initial assessment, and there was no evidence of root resorption or periapical lesion.

## 3 CONCLUSION

The present case report highlights the efficacy and favorable outcome of auto‐transplantation as a viable treatment option for replacing missing or non‐restorable permanent molar teeth. Factors such as asepsis, atraumatic surgical technique, preservation of the periodontal ligament vitality, minimal extraoral time, and optimal occlusion were crucial in ensuring the success of the procedure. The 1‐year follow‐up demonstrated stable occlusion and function in this patient, emphasizing the long‐term efficacy of mature third molar transplantation in restoring both esthetics and function, providing a promising alternative to traditional tooth replacement methods.

## 4 DISCUSSION

ATT is a complex procedure that involves the extraction and repositioning of a tooth from one site to another in the same patient. This case report describes a successful case of ATT in a 66‐year‐old woman with a non‐restorable mandibular right second molar with a mesiodistal crack in the root. The patient's preference for preserving her natural dentition and avoiding tooth extraction made ATT an appealing treatment option. Since the adjacent third molar was not in contact with any opposing tooth in the occlusion, it was suitable for transplantation.

Infection of the root canal system is a critical factor contributing to development of inflammatory root resorption after ATT. Therefore, pulp extirpation is imperative within 1–2 weeks after ATT of closed‐apex teeth to avoid pulpal infection and subsequent periradicular inflammation and inflammatory root resorption.^11^ This is justified by the fact that only 15% of the teeth with closed apices, versus 96% of the teeth with open apices, are revitalized after ATT.^12^ Therefore, for the present case, root canal treatment was performed with a bioceramic sealer. In contrast to a previous case report that emphasized on retrograde root canal treatment to minimize unnecessary tooth structure destruction,^13^ orthograde treatment was performed in the present case to reduce extraoral time, preserve the tooth, minimize the risk of damage to periodontal fibers, decrease the risk of infection and contamination, and also due to the short length of the third molar's root. Application of a root‐end filling biomaterial plays a crucial role in the success of the procedure.^14^ An appropriately applied root‐end filling material can effectively seal the communication paths and prevents subsequent inflammation of the periradicular tissues. Also, hermetic sealing of the root end minimizes the risk of microbial contamination and subsequent inflammation of the periradicular tissue. Thus, the entire working length of the canals was filled with bioceramic sealer in the present case.

According to Zachrisson et al.,^15^ the following critical factors must be taken into account in the surgical procedure for ATT: the donor tooth extra‐alveolar time, and both the skills and experience of the surgeon, which are highly important. Traumatization of the PDL should be avoided, since it can lead to ankylosis. The presence of sufficient space at the mesial and distal sides of the transplanted tooth, elimination of occlusal interferences (oscillating contacts) between the transplanted tooth and the opposing teeth during the first 2 months, and physiological mobility during the fixation period are important prerequisites for the success of treatment. In this case report, the surgical procedure was completed in less than 5 min. Due to the speed of the operation, apical root resection was omitted, prioritizing efficiency. Immediately after extraction of the mandibular second molar, the third molar was transplanted.

Numerous studies have reported high success and survival rates for ATT.^16^ However, the success of ATT is influenced by factors such as the patient's age, donor tooth type, and surgical technique. In comparison, dental implants have a high success rate, but the procedure is more complex and may require additional surgery.^16^

The favorable results of ATT obtained in the present case may be attributed to factors such as optimal asepsis during the surgical procedure, atraumatic surgical extraction and replacement, preservation of cellular vitality of the PDL, minimal extraoral time, good occlusion, adequate fixation, and filling and sealing of the apex with a biocompatible root‐end filling material. The patient was asymptomatic at the 1‐year follow‐up with stable function and occlusion, highlighting the optimal efficacy of mature third molar transplantation as an effective and promising approach for replacement of a missing or non‐restorable permanent molar tooth to restore both esthetics and function.

## AUTHOR CONTRIBUTIONS

**Mohsen Aminsobhani:** Conceptualization; investigation; methodology; writing – review and editing. **Maryam Babaahmadi:** Conceptualization; investigation; methodology; validation; writing – original draft.

## CONFLICT OF INTEREST STATEMENT

The authors deny any conflict of interest.

## ETHICS STATEMENT

For clinical cases, the local ethics committee considers that the patient's consent is sufficient.

## CONSENT

Written informed consent was obtained from the patient to publish this report in accordance with the journal's patient consent policy.

## Data Availability

The data supporting the findings of the present study are available from corresponding author upon request.
